# Identifying *FecB* genotypes in the muscle from sheep breeds indigenous to Xilingol, and establishment of a *Taq*Man real‐time PCR technique to distinguish *FecB* alleles

**DOI:** 10.1002/fsn3.2853

**Published:** 2022-03-30

**Authors:** Liang Guo, Chun‐Dong Li, Guo‐Qiang Liu, Jian‐Xing Luo, Wei‐Liang Xu, Yuan‐Sheng Guo

**Affiliations:** ^1^ Xilin Gol Food Testing and Risk Assessment Center Xilingol Vocational College Xilin Gol Institute of Bioengineering Xilinhot China

**Keywords:** *FecB*, PCR‐RFLP, SNPs, *Taq*Man

## Abstract

The muscle from Xilingol indigenous sheep breeds are famous in China, and the *FecB* genotype in this population remains uncharacterized. In this study, SNPs in the *FecB* locus were investigated by pyrosequencing, and an optimized PCR‐RFLP technique was generated to identify SNPs. In addition, an efficient technique for high‐throughput identification of SNPs in *FecB* was optimized using *Taq*Man real‐time PCR and breed‐conservative primers and SNP‐specific probes. By genotyping the *FecB* locus in the muscle of Xilingol indigenous sheep breeds using a novel *Taq*Man real‐time PCR assay, our study has generated the groundwork for the authentication of Xilingol mutton based on the specific gene and the prolificacy‐oriented breeding of Xilingol sheep using marker‐assisted selection strategies in the future.

## Graphical abstract

First, this study is the first to investigate the SNPs of *FecB* gene in mutton from Xilingol indigenous sheep breeds. Second, in order to develop an efficient high‐throughput technique for identification of the mutations in the *FecB* gene in mutton, a novel TaqMan real‐time PCR technique was designed inferred from breed‐conservative primers and SNP‐specific probes.

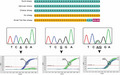

## 1 INTRODUCTION

The Booroola locus (*FecB*) carries a dominant autosomal mutation that is responsible for the hyperprolific characteristics of Booroola ewes, which was first discovered in the Australian Merino breed (Davis, 2004). The high fecundity of Booroola ewes results from the *FecB* mutation in the *bone morphogenetic protein receptor 1B* (*BMPR‐1B*) gene, which encodes a transforming growth factor β gene (TGFβ) (Mulsant et al., 2001; Souza et al., 2001). The *FecB* mutation leads to higher ovulation rate by affecting follicular fluid and ovarian vein serum (Guo et al., 2018). The point mutation (A turn to G) at base 746 of the coding region of *BMPR‐1B,* changing a glutamine to an arginine, is associated with the hyperprolific profile of Booroola ewes (Souza et al., 2001; Wilson et al., 2001). Thus, the *FecB* mutation has become one of the candidates for breeding sheep for high prolificacy using marker‐assisted selection (Chen et al., 2015; Hua & Yang, 2009).

The Xilingol grassland in China is a natural grazing area, it is known for its muscle and for its high‐quality pollution‐free and free‐range sheep. The average annual output in Xilingol is more than 10 million sheep. The indigenous sheep breeds from Xilingol are Sunit sheep, Ujimuqin sheep, and Chahar sheep. Despite the thriving sheep farming in the Xilingol region, SNPs in the *FecB* locus remain unknown in the above three breeds. The hyperprolific effect of the *FecB* mutation can serve as a genetic marker in marker‐assisted selection strategies to increase litter size of sheep (Chen et al., 2015; Wang et al., 2018). SNPs in the *FecB* locus are usually identified using PCR‐RFLP (Chu et al., 2007; Mahdavi et al., 2014), PCR‐SSCP (Chu et al., 2011; Mulsant et al., 2001), pyrosequencing (Souza et al., 2001), ARMS PCR (Ahlawat et al., 2014), the KASPar method (Wang et al., 2018), and *Taq*Man real‐time PCR (Woodward, 2014). In general, real‐time PCR using *Taq*Man probes is regarded as the most rapid and high‐throughput assay when using probes that are specific for the breed of interest.

The objective of this study was to develop a novel *Taq*Man real‐time PCR protocol that can be used to identify SNPs in the *FecB* locus in the muscle from Xilingol indigenous sheep breeds using SNP‐specific probes targeting the *FecB* locus. The assay with different *Taq*Man probes can be further used for the authentication of Xilingol mutton based on the specific gene and genotyping sheep from the Xilingol region for marker‐assisted selection.

## 2 MATERIALS AND METHODS

### 2.1 Muscle DNA extraction

A total of 150 muscle samples belonging to five pure breeds of sheep (Sunit sheep, Ujimuqin sheep, Chahar sheep, Hu sheep, and Small Tail Han sheep) were analyzed. Sheep breeds indigenous to the Xilingol region are Sunit sheep, Ujimuqin sheep, and Chahar sheep, and the muscle samples for these breeds were collected from the conservation farm of Xilingol Animal Improving Station in China. Muscle for Hu sheep and Small Tail Han sheep were obtained from the conservation farms of Linan city and Heze city in China, respectively. Genomic DNA was extracted using the Genomic DNA Kit (Tansgen, Beijing, China), and the concentration and integrity of the DNA samples were assessed using spectrophotometer and agarose gel electrophoresis analyses, and only samples that met the requirements (DNA concentration ≥100 ng/μl and no degradation of DNA band in gel) for conventional and real‐time PCR protocol were kept. The experiments in this study were performed following the ethical guidelines and regulations of the Laboratory Animal Welfare and Ethics Committee of China (CALAS).

### 2.2 Pyrosequencing and PCR‐RFLP

The primers (LP1 and RP1) for pyrosequencing and PCR‐RFLP were generated by Ruibiotech Company (Beijing, China) using the sequences described by Wilson et al. (2001) (Table 1). Moreover, optimized primers (LP2 and RP2) were developed to identify PCR‐RFLP results easier (Table 1). Conventional PCR reaction mixtures (20 μl) contained 10 μl PCR SuperMix (Tansgen), 1 μl LP (10 μmol/l), 1 μl RP (10 μmol/l), 1 μl template (100 ng/μl), and 7 μl ddH_2_O. The PCR reactions were performed under the following program: 5 min at 94℃, 35 cycles of 30 s at 94℃, 30 s at corresponding annealing temperature (Table 1) and 60 s at 72℃, and 10 min at 72℃ (ABI2720; Applied Biosystems, Waltham, MA, USA). The amplified PCR products were gel purified using the MiniBEST Agarose Gel DNA Extraction Kit (TaKaRa, Dalian, China), and sequenced by Ruibiotech Company (Beijing, China). The purified DNA fragments were digested using *AvaII* for 4.5 h at 37℃ following the manufacturer's protocol (New England Biolabs, Ipswich, MA, USA).

**TABLE 1:** The primers and probes that were used for conventional and real‐time PCR

Primers Probes	Sequence (5'–3')	Annealing Temp.
LP1	CCAGAGGACAATAGCAAAGCAAA	58℃
RP1	CAAGATGTTTTCATGCCTCATCAACAGGTC	
LP2	TTTAACAGGTCCAGAGGACAATAGCAAAGCAAA	61℃
RP2	AATACAGACTTATACTCACCCAAGATGTTTTCATGCCTCATCAACAGGTC	
LP3	AGCTGTGAAAGTGTTCTTCA	60℃
RP3	GATGTTTTCATGCCTCATCA	
Probe‐A	FAM‐CACCGTCTGATATATTTCTGTCTCTCGG‐TAMRA	
Probe‐G	HEX‐CACCGTCCGATATATTTCTGTCTCTCGG‐TAMRA	

### 2.3 *TaqMan* Real‐time PCR

*Taq*Man real‐time PCRs were carried out using primers (LP3 and RP3) and probes (Probe‐A and Probe‐G) (Table 1) designed to identify SNPs in the *FecB* locus. The primers and probes were synthesized by Ruibiotech (Beijing, China). *Taq*Man real‐time PCR reaction mixtures (20 μl) contained 10 μl Probe qPCR SuperMix (Tansgen), 1 μl LP3 (10 μmol/l), 1 μl RP3 (10 μmol/l), 1 μl Probe‐A (10 μmol/l), 1 μl Probe‐G (10 μmol/l), 1 μl template (100 ng/μl), and 5 μl ddH_2_O. The real‐time PCR reactions were performed under the following program: 30 s at 94℃, 40 cycles of 5 s at 94℃ and 31 s at 60℃ (FTC‐3000P; Funglyn Biotech Inc., Ontario, ON, Canada).

## 3 RESULTS AND DISCUSSION

### 3.1 Amplification and sequencing of the *FecB* locus in the muscle from Mongolian sheep breeds

**FIGURE 1:**
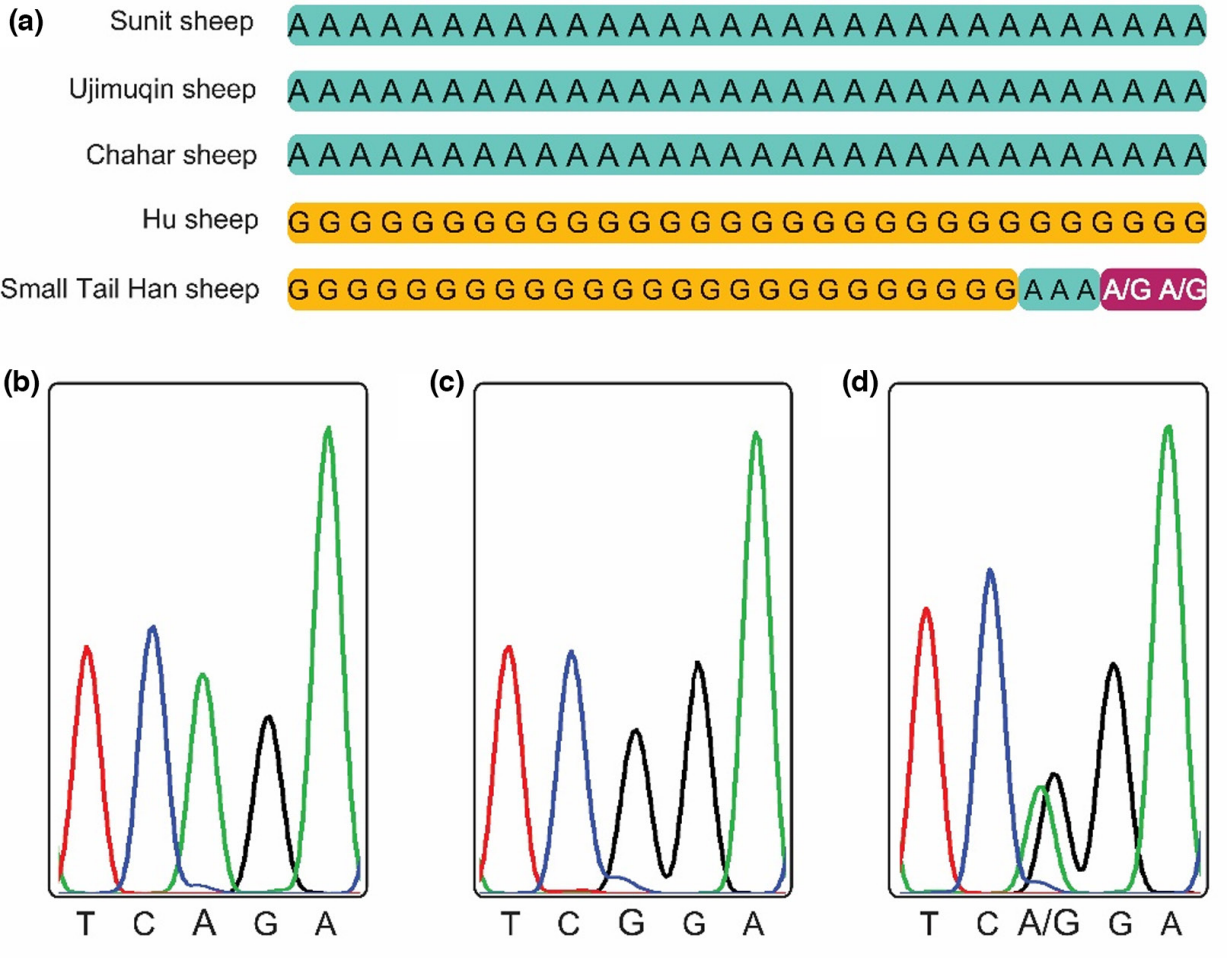
The *FecB* locus was sequenced in sheep breeds indigenous to the Xilingol region, and the Hu and Small Tail Han breeds. SNPs at the *FecB* locus are shown from 30 sheep per breed (a). The genotypes of SNP‐A (b), SNP‐G (c), and SNP‐A/G (d) are displayed for the corresponding fluorescence signals of pyrosequencing

The *FecB* locus has two alleles in mutton, where A is the wild‐type nucleotide and G is the mutant nucleotide. For the *FecB* locus, the G variant leads to a change in amino acid from glutamine to arginine (Souza et al., 2001). The presence of the *FecB* mutation has been investigated in a few prolific breeds, such as Booroola Merino (Mulsant et al., 2001), Indian Bonpala sheep (Roy et al., 2011), Iranian Kalehkoohi sheep (Mahdavi et al., 2014), Chinese Hu sheep (Chu et al., 2011; Guan et al., 2007), Chinese Merino prolific meat strain (Guan et al., 2007), and Chinese Small Tail Han Sheep (Chu et al., 2004, 2007). Nevertheless, the mutations in the *FecB* gene remain unknown in the muscle from three Xilingol indigenous sheep breeds. We analyzed the nucleotide sequences of the *FecB* locus by pyrosequencing from 30 animals each of the three Xilingol indigenous sheep breeds. After amplifying the *FecB* region using conventional PCR, the nucleotide sequences of the *FecB* locus were sequenced. Sunit sheep, Ujimuqin sheep, and Chahar sheep all had the A variant at base 746 of the coding region of *BMPR‐1B* (Figure 1a). Interestingly, all Hu sheep individuals had the G variant in the *FecB* locus (Figure 1a). Of the 30 individuals that were sampled from the Small Tail Han sheep, 25 had the G variant, three had the A variant, and two had the A/G heterozygous variant (Figure 1a). Therefore, the breeds indigenous to Xilingol had the wild‐type A variant of the *FecB* locus, while Hu and Small Tail Han sheep had the mutant G nucleotide.

Our results are consistent with previous studies that have investigated the *FecB* locus in Hu and Small Tail Han sheep (Hua & Yang, 2009). Moreover, the G mutant nucleotide in the *FecB* locus was directly linked to the high prolificacy in Hu and Small Tail Han sheep breeds (Chu et al., 2004, 2011; Guo et al., 2018; Wang et al., 2018). The National Commission of Animal Genetic Resources of China reported an annual breeding rate of 113% for Sunit sheep, 113% for Ujimuqin sheep, 126.4% for Chahar sheep, 277.4% for Hu sheep, and 267.1% for Small Tail Han sheep (China National Commission of Animal Genetic Resources, 2011; Xu et al., 2016). We had hypothesized that the A nucleotide in the *FecB* locus in the Xilingol indigenous sheep breeds promoted their fecundity to be lower compared to Hu and Small Tail Han sheep. Furthermore, the genetic relationships of Sunit sheep, Ujimuqin sheep, Hu sheep, and Small Tail Han sheep suggest that they evolved from a Mongolian breed of sheep (China National Commission of Animal Genetic Resources, 2011; Xu et al., 2016). We speculated that the grazing regime of Mongolian sheep in the Xilingol prairie in Northern China was selected for its low prolificacy, allowing these breeds to survive the winter, and farmers in the Shangdong and Zhejiang provinces in Western China reared sheep in barns and artificially selected sheep for their high prolificacy. Eventually, the Mongolian sheep with low prolificacy in the Xilingol prairie gradually evolved to form Sunit sheep in the East and West Sunit banner, and Ujimuqin sheep breed in East and West Ujimuqin banner, and these with high prolificacy formed Hu sheep breed in Zhejiang province, and Small Tail Han sheep breed in Shandong province.

### 3.2 Optimization of PCR‐RFLP to identify *FecB* alleles

**FIGURE 2:**
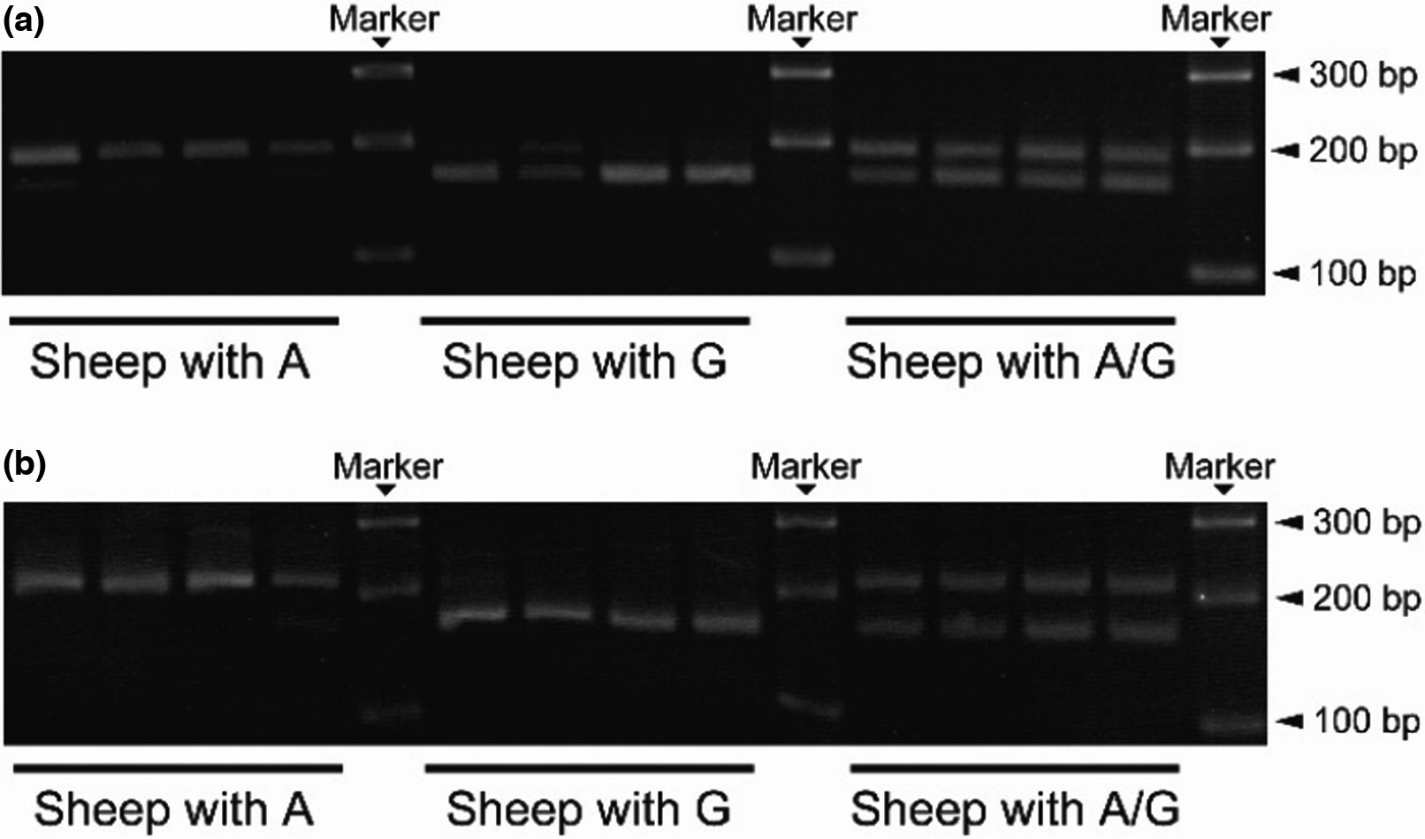
The SNPs at base 746 of the *FecB* locus were analyzed by PCR‐RFLP using published primers (LP1 and RP1) (a) and new primers that generate longer fragments to be more identifiable (LP2 and RP2) (b). Four samples each from individuals known to carry the A, G, and A/G variants of *FecB* were used as templates for PCR‐RFLP

In order to differentiate between the A and G variants of *FecB*, we used a published pair of primers (LP1 and RP1) to amplify a 190‐bp fragment containing the *FecB* locus (Davis et al., 2002; Wilson et al., 2001). A restriction enzyme site was introduced into the RP1 primer, which led to the amplification of PCR fragments containing the *AvaII* restriction site of the *FecB* locus carrying the G variant (G|GACC). Sequences from four sheep with the A nucleotide, four sheep with the G nucleotide, and four sheep with the A/G nucleotide were amplified and restriction digested using *AvaII* to verify the introduction of the cut site (Figure 2a). Individuals carrying the A or G nucleotide had a 190‐bp or 160‐bp band, respectively (Figure 2a). Fragments from the heterozygous individuals were digested into both 190‐ and 160‐bp bands (Figure 2a). Taken together, the new primers were effective in distinguishing SNPs in the *FecB* locus after restriction enzyme digestion.

PCR‐RFLP is regarded as a simple method for identifying SNPs. The above PCR‐RFLP approach using the *AvaII* restriction site has been used previously to genotype prolific sheep (Chu et al., 2007; El‐Seedy et al., 2017; Ganai et al., 2012; Gootwine et al., 2008; Kumar et al., 2008; Mahdavi et al., 2014; Xu et al., 2010). Nevertheless, improvements were needed to better screen for prolific sheep. First, conventional DNA ladders cannot discriminate the 190‐ and 160‐bp bands, which can be difficult to distinguish in the agarose gel. Second, the 190‐ and 160‐bp bands can sometimes appear as one band in gels with low concentration of agarose. Thus, we developed optimized primers (LP2 and RP2) for PCR‐RFLP to identify the *FecB* alleles (A, G, or A/G). RP2 was designed to introduce a point mutation that results in a 220‐bp fragment containing the *AvaII* restriction site (G|GACC) if the fragment contains the G nucleotide. DNA from four individuals each carrying the A, G, and A/G variant in the *FecB* locus were used to test the effectiveness of the LP2 and RP2 primers (Figure 2b). PCR products that were restriction digested using *AvaII* yielded 220‐ and 170‐bp bands for individuals carrying the A or G nucleotides, respectively (Figure 2b). Individuals that were heterozygous yielded both 220‐ and 170‐bp bands (Figure 2b). These results validate the new primer pair, which allowed for easier differentiation of the two genotypes, as it is easier to separate 220‐ and 170‐bp bands, compared to 190 and 160 bp.

### 3.3 Development and validation of a *TaqMan* real‐time PCR technique to distinguish the *FecB* alleles

While PCR‐RFLP can be carried out using basic laboratory equipment to relatively high levels of success, distinguishing the relative position of the amplified nucleotide sequence in an agarose gel can be prone to error. Error can be introduced through the difference in the quantity of DNA used for restriction digest, the digestion efficiency of the restriction enzyme, the length of digestion, the concentration of the agarose gel, and the volume of sample loaded during electrophoresis. *Taq*Man real‐time PCR provides a more reliable alternative for genotyping SNPs (Gaedigk et al., 2015; Schleinitz et al., 2011; Woodward, 2014). In particular, real‐time PCR results can be observed without electrophoresis, and can be used in a high‐throughput manner.

**FIGURE 3:**
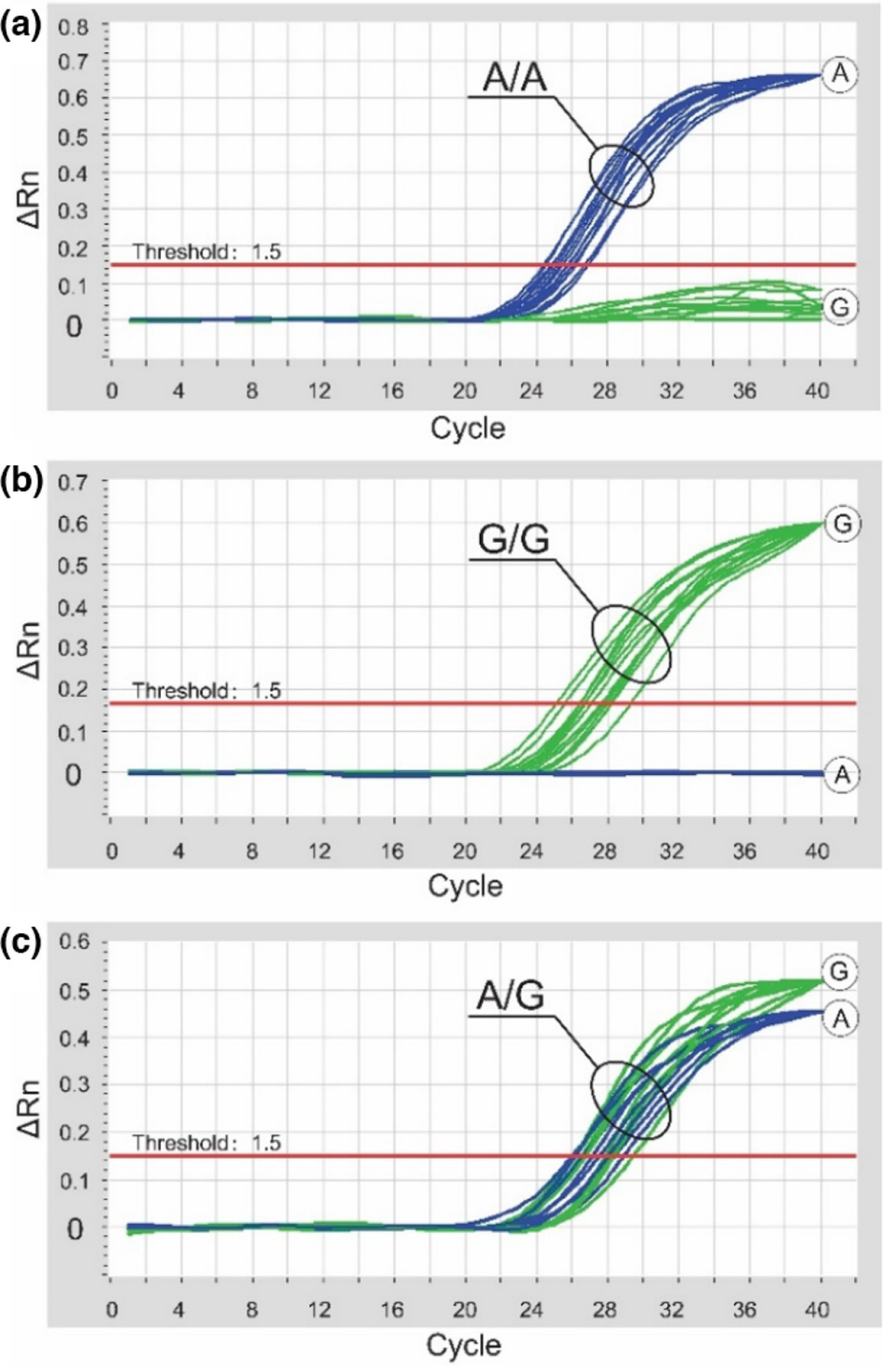
*Taq*Man real‐time PCR to identify the A variant (a), G variant (b), and heterozygous A/G variant (c) of the *FecB* locus. The homozygous variants were confirmed using 10 sheep with known genotypes. FAM or HEX were simultaneously identified in the amplification plots of eight heterozygous sheep in (c)

For these reasons, we sought to develop an efficient technique for high‐throughput identification of the *FecB* alleles using *Taq*Man real‐time PCR with primers (LP3 and RP3) and SNP‐specific probes (Probe‐A and Probe‐G) (Table 1). Samples from 10 individuals each carrying the A and G variant and eight sheep carrying the A/G variant were used to validate the probes (Figure 3). The A‐specific *Taq*Man probe was labeled with FAM (fluorophore) and TAMRA (quencher), and the G‐specific *Taq*Man probe was labeled with HEX (fluorophore) and TAMRA (quencher). This facilitated the simultaneous identification of the A and G variants by detecting the fluorophores FAM and HEX. Probe‐A successfully amplified the A nucleotide, and Probe‐G successfully amplified the G nucleotide using DNA from homozygous individuals (Figure 3a,b). In addition, Probe‐A and Probe‐G amplified fragments from the A/G heterozygous individuals (Figure 3c). Taken together, the duplex *Taq*Man real‐time PCR was effective and robust for identifying SNPs in the *FecB* locus.

## 4 CONCLUSIONS

First, the *FecB* locus was genotyped in the muscle from three Xilingol indigenous sheep breeds. Sunit sheep, Ujimuqin sheep, and Chahar sheep all carried the wild‐type variant of *FecB*. Then, the well‐established PCR‐RFLP method was used to detect SNPs in the *FecB* locus, and an optimized PCR‐RFLP technique was developed. Lastly, we established an efficient technique for high‐throughput identification of *FecB* alleles using *Taq*Man real‐time PCR with SNP‐specific probes. In conclusion, the *FecB* alleles of the muscle from sheep breeds indigenous to the Xilingol region were identified and a robust and high‐throughput *Taq*Man real‐time PCR technique was established to authenticate the muscle from Xilingol sheep.

## CONFLICT OF INTEREST

All authors declare no conflict of interest.

## ETHICAL APPROVAL

This study does not involve any human or animal testing.

## Data Availability

The data that support the findings of this study are available from the corresponding author upon reasonable request.
